# Supplementary material for: BIK1 protein homeostasis is maintained by the interplay of different ubiquitin ligases in immune signaling
Source: Nat Commun. 2023 Aug 2;14:4624. doi: 10.1038/s41467-023-40364-0 (PMC10397244; doi:10.1038/s41467-023-40364-0)
Supplement: Supplementary file 1 — Supplementary Information [file 41467_2023_40364_MOESM1_ESM.pdf]

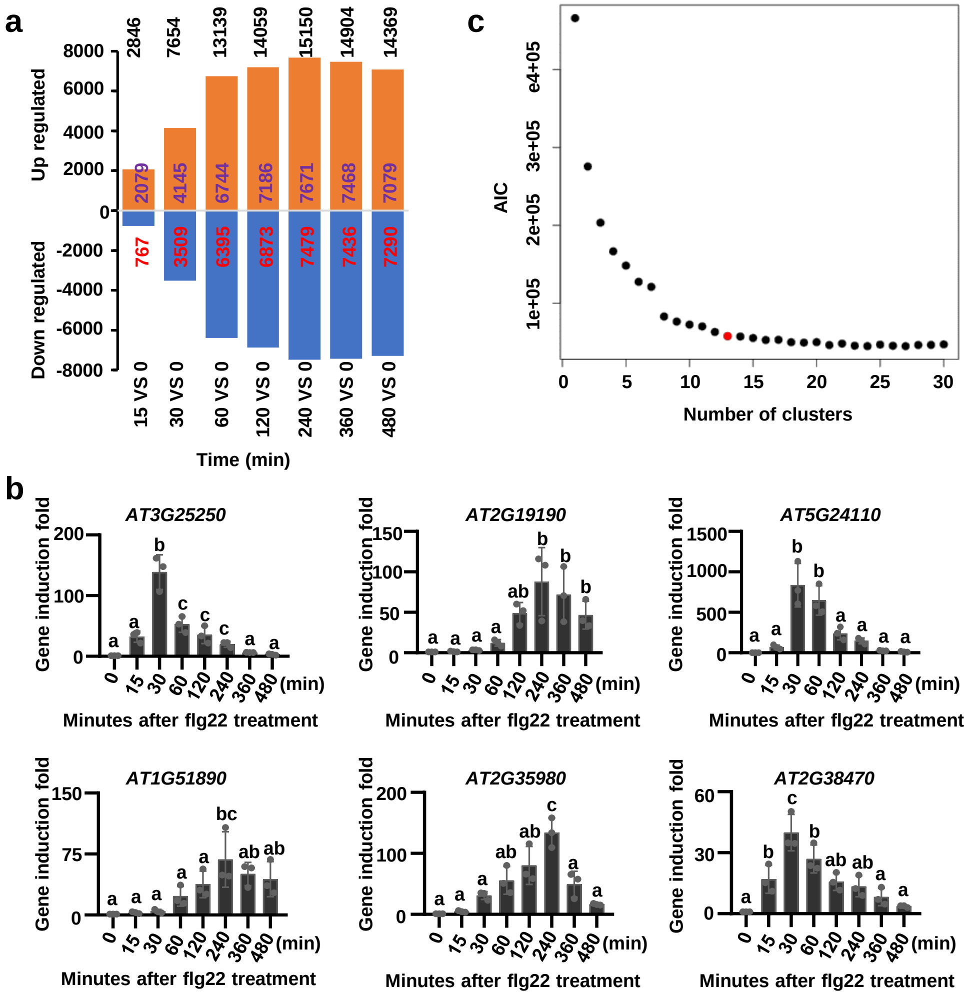


**Supplementary Fig. 1 RNA-seq analysis of differentially expressed genes in response to persistent flg22 treatment. a**, The number of differentially expressed genes (DEGs) after flg22 treatment. DEGs were identified by comparison with time 0 via the R package DESeq2 [$\log_2$(fold change, FC) > 1, $P_{adj} < 0.05$]. Yellow columns represent the up-regulated genes and blue ones indicate the down-regulated genes. **b**, Verification of gene expression by RT-qPCR. *GAPC* was used as an internal control. Values are the means ± SD of three biological repeats using independent seedling samples grown under the same conditions. Different letters denote significance difference (One-way ANOVA, $P$ < 0.05).). Exact $P$ value are < 0.0001 (*AT3G25250*); 0.0003 (*AT2G19190*); < 0.0001 (*At5G24110*); 0.0008 (*AT1G51890*); < 0.0001 (*AT2G35980*); < 0.0001 (*AT2G38470*). Source data are provided as a Source Data file (Supplementary dataset 4). **c**, AIC value changes with the number of gene clusters ($K$).

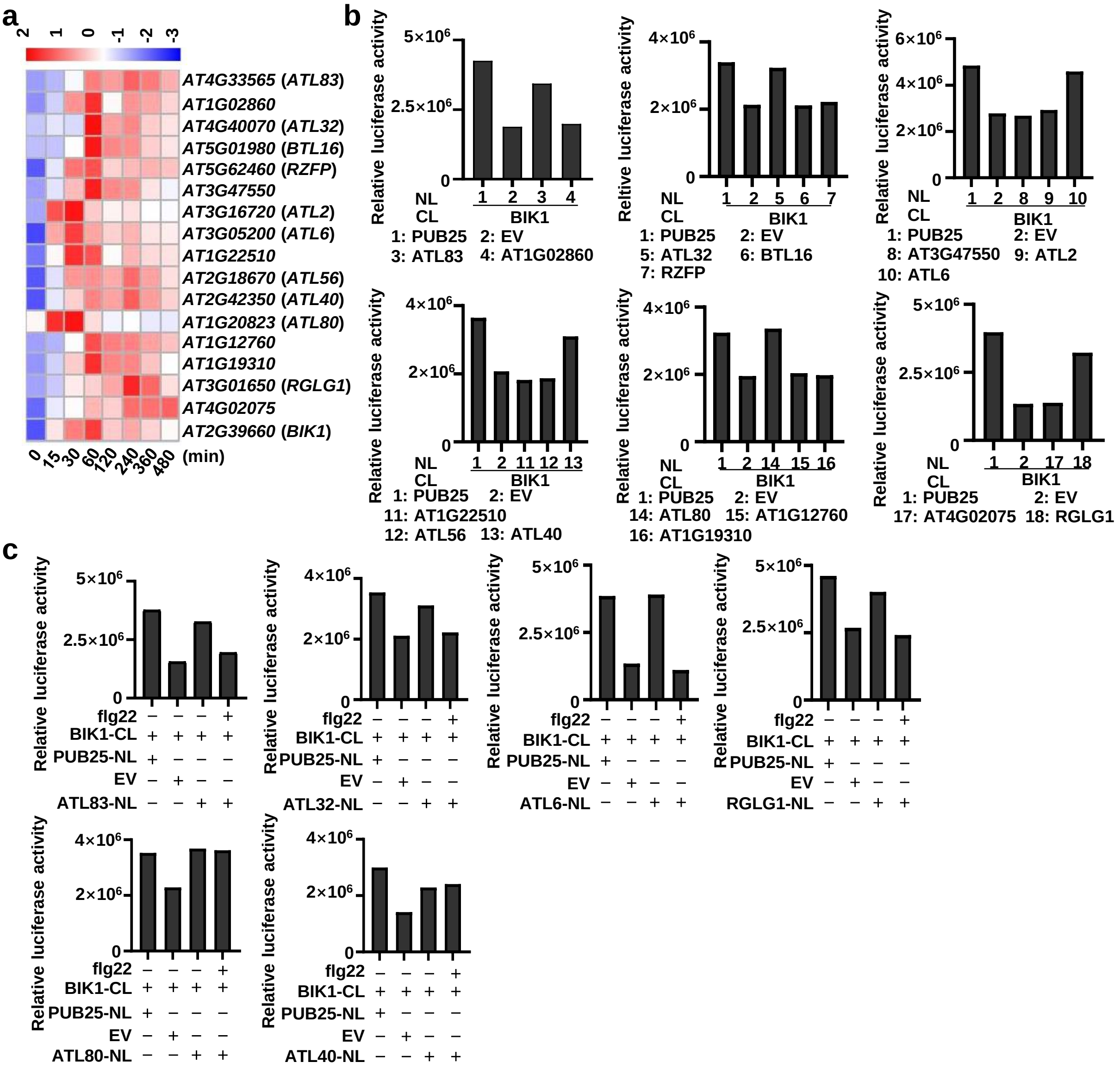


**Supplementary Fig. 2 Screening of E3 ubiquitin ligases that interact with BIK1. a**, Expression heatmap of genes (with the expression pattern #2) encoding E3 ubiquitin ligases that were predicted to be localized to the plasma membrane. The heatmap was based on the FPKM values of three biological replicates. **b**, Screening of E3 ubiquitin ligases that interact with BIK1 by split-luciferase complementation assays. *Nicotiana benthamiana* leaves were infiltrated with *Agrobacterium tumefaciens* (GV3101) harboring CLuc-BIK1 and E3-NLuc. luminescence images were captured using a CCD imaging system (Fusion FX7, Vilber, Marne-la-Vallée, France), the relative luciferase activity in leaves was quantified using kuant 1.5 (Vilber, France) software, values are means of two biological repeats using independent plants grown and inoculated under the same conditions. PUB25-NLuc and empty NLuc vector (EV) were used as positive and negative controls, respectively. **c**, The association of several ubiquitin ligases with BIK1 is reduced upon flg22 treatment as determined by split-luciferase complementation assays. Six ubiquitin ligases that were shown to be associated with BIK1 in (**b**) were expressed together with BIK1 as described in (**b**). The *N. benthamiana* leaves were treated with or without 5 μM flg22 for 30 min before harvesting. Split-luciferase complementation assays were performed as in (**b**).

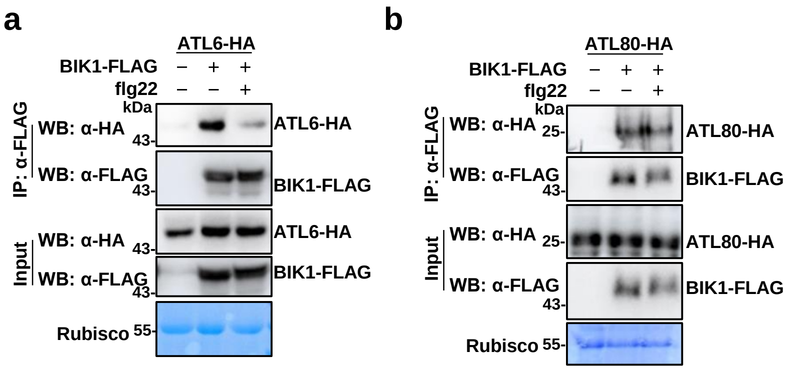


**Supplementary Fig. 3 The association of BIK1 with ATL6 or ATL80 as assayed by co-IP. a** and **b**, BIK1-FLAG was co-expressed with ATL6-HA (**a**) or ATL80-HA (**b**) in Arabidopsis protoplasts. The protoplasts were treated with or without 2.5 µM flg22 for 30 min. BIK1-FLAG was immunoprecipitated with anti-FLAG antibodies, the associated ATL6-HA or ATL80-HA proteins were detected by immunoblotting with anti-HA antibodies. Images shown are representative of at least two independent experiments.

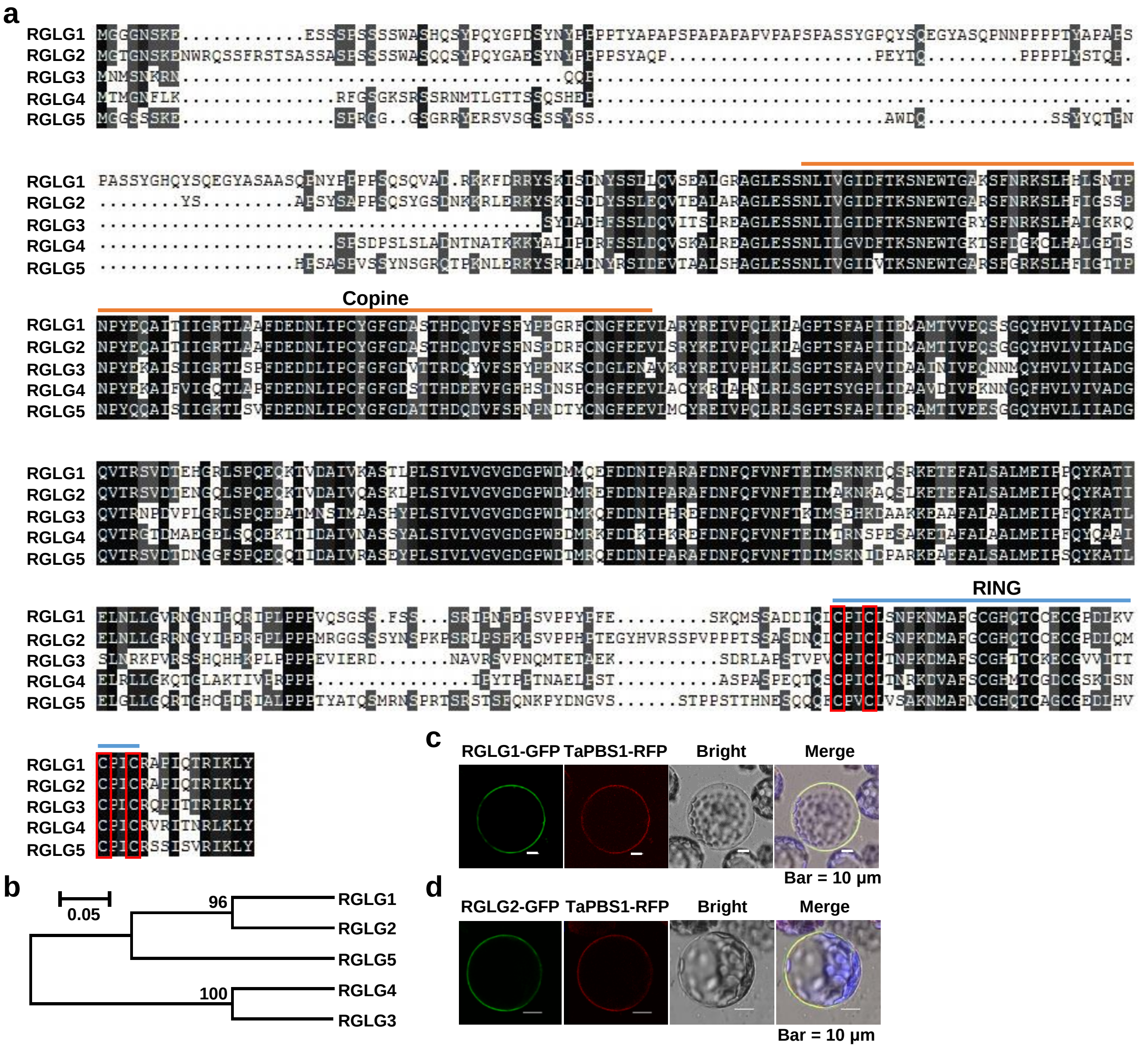


**Supplementary Fig. 4 Characterization of RGLG family proteins. a,** Amino acid sequence alignment of Arabidopsis RGLG1-RGLG5. Identical and similar amino acid residues were displayed on black and gray backgrounds, respectively. Four conserved Cys sites that chelate $Zn^{2+}$ in the RING domain were highlighted in red boxes. **b,** Phylogenetic analysis of RGLGs. A neighbor-joining phylogenetic tree was constructed based on the deduced amino acid sequences of RGLGs using MEGA5.0.3 software. **c** and **d,** RGLG1 and RGLG2 are localized to the plasma membrane. RGLG1-GFP or RGLG2-GFP was co-expressed with TaPBS1-RFP in Arabidopsis protoplasts. Green and red fluorescent proteins were visualized via confocal microscopy. Scale bar = 10 µm. Images shown are representative of at least two independent experiments.

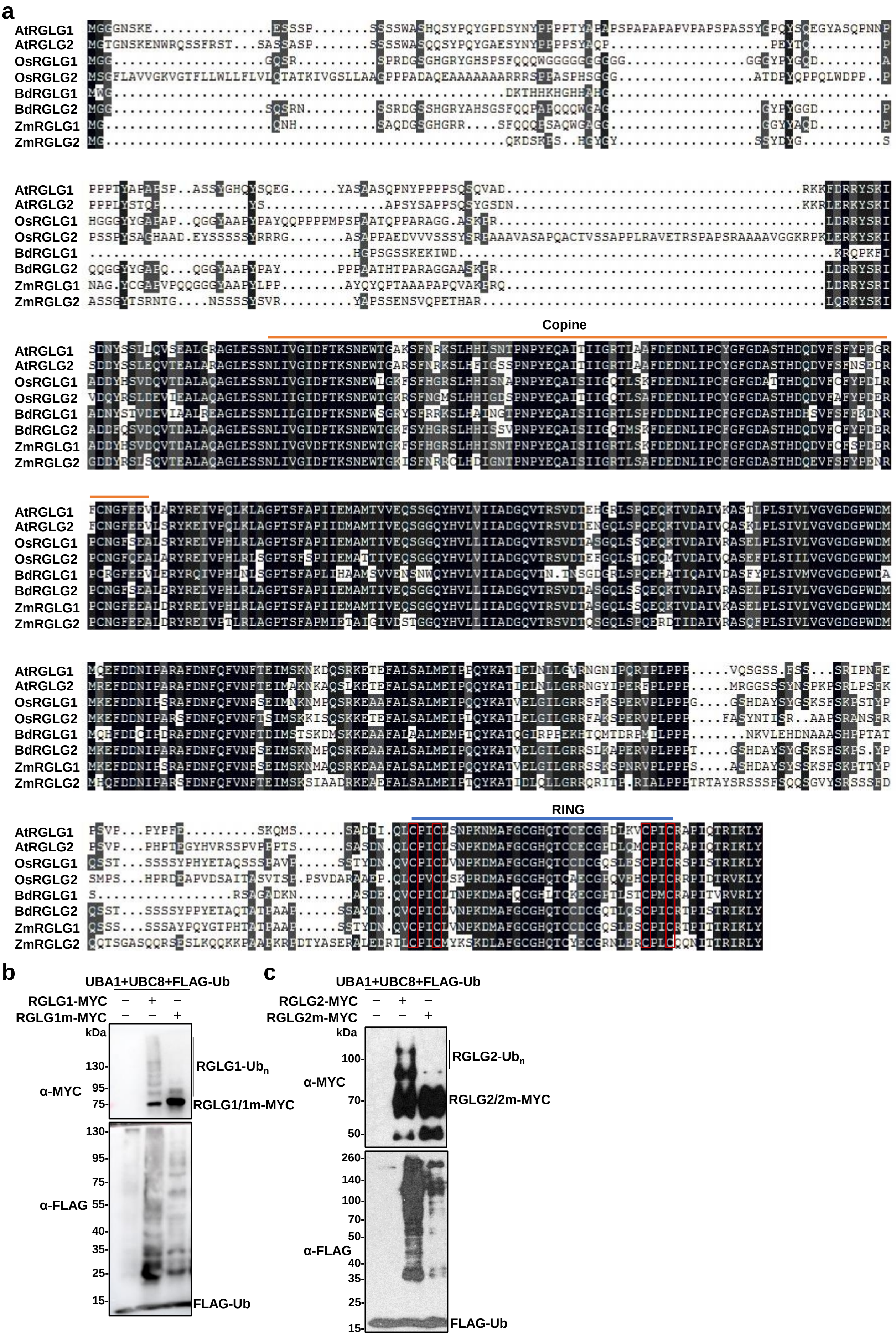
a
AtRGLG1
AtRGLG2
OsRGLG1
OsRGLG2
BdRGLG1
BdRGLG2
ZmRGLG1
ZmRGLG2
Copine
RING
b
UBA1+UBC8+FLAG-Ub
RGLG1-MYC − + −
RGLG1m-MYC − − +
kDa
130-
95-
75-
α-MYC
RGLG1-Ubn
RGLG1/1m-MYC
130-
95-
75-
55-
40-
35-
25-
15-
α-FLAG
FLAG-Ub
c
UBA1+UBC8+FLAG-Ub
RGLG2-MYC − + −
RGLG2m-MYC − − +
kDa
100-
70-
50-
α-MYC
RGLG2-Ubn
RGLG2/2m-MYC
260-
140-
100-
70-
50-
40-
35-
25-
15-
α-FLAG
FLAG-Ub

**Supplementary Fig. 5 RGLG1/2 have ubiquitin ligase activity *in vitro*. a**, Amino acid sequence alignment of Arabidopsis RGLG1/2 with putative RGLG1/2 orthologs from rice (OsRGLG1/2), maize (ZmRGLG1/2), and *Brachypodium distachyon* (BdRGLG1/2). Identical and similar amino acid residues were displayed on black and gray backgrounds, respectively. Four conserved Cys sites that chelate Zn2+ in the RING domain were highlighted in red boxes. **b** and **c**, The ubiquitin ligase activity of RGLG1m and RGLG2m were largely impaired. AtUBA1, AtUBC8, His-FLAG-Ub, RGLG1/RGLG1m-MYC (**b**), or RGLG2/RGLG2m-MYC (**c**) were co-expressed in *E. coli*, the bacterial lysates were subjected to immunoblotting analysis with anti-MYC or anti-FLAG antibodies for detecting the autoubiquitination of RGLG1/2 variants. Images shown are representative of three independent experiments.

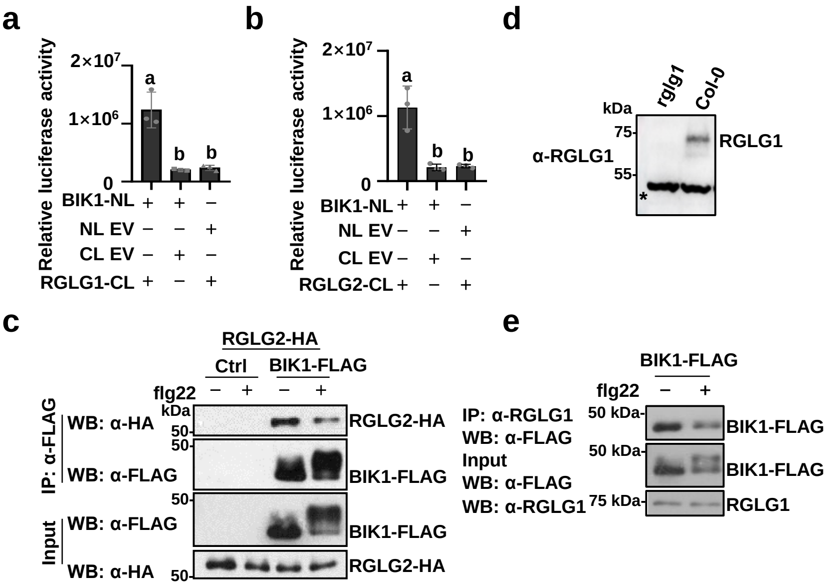


**Supplementary Fig. 6 RGLG1/2 associate with BIK1. a** and **b**, BIK1 associates with RGLG1/2 as assayed by split-luciferase complementation. BIK1-NLuc and RGLG1/2-CLuc were expressed in *N. benthamiana*. Empty NLuc or CLuc vector (EV) was used as negative controls. Relative luciferase activity was quantified and the values are means ± SD ($n$ = 3 leaves from three independent biological repeats). Different letters denote significance difference (one-way ANOVA, $P < 0.05$). $P = 0.0006$ (**a**), $P = 0.0017$ (**b**). Source data are provided as a Source Data file (Supplementary dataset 4 and Supplementary Table 1). **c**, RGLG2 associate with BIK1 as assayed by co-IP. RGLG2-HA and BIK1-FLAG were co-expressed in Arabidopsis protoplasts. The protoplasts were treated with or without 2.5 µM flg22 for 30 min. BIK1-FLAG was immunoprecipitated with anti-FLAG antibodies, the associated RGLG1/2-HA proteins were detected by immunoblotting with anti-HA antibodies. Images shown are representative of three independent experiments. **d**, Verification of the specificity of anti-RGLG1 antibodies. Total proteins were extracted from Col-0 and *rglg1* mutant. Asterisks indicate nonspecific bands. **e**, RGLG1 associates with BIK1 as assayed by co-IP using anti-RGLG1 antibodies. BIK1-FLAG was expressed in Arabidopsis (Col-0) protoplasts. The protoplasts were treated with or without 2.5 µM flg22 for 30 min. IPs were performed using anti-RGLG1 antibodies. **d** and **e**, images shown are representative of at least two independent experiments.

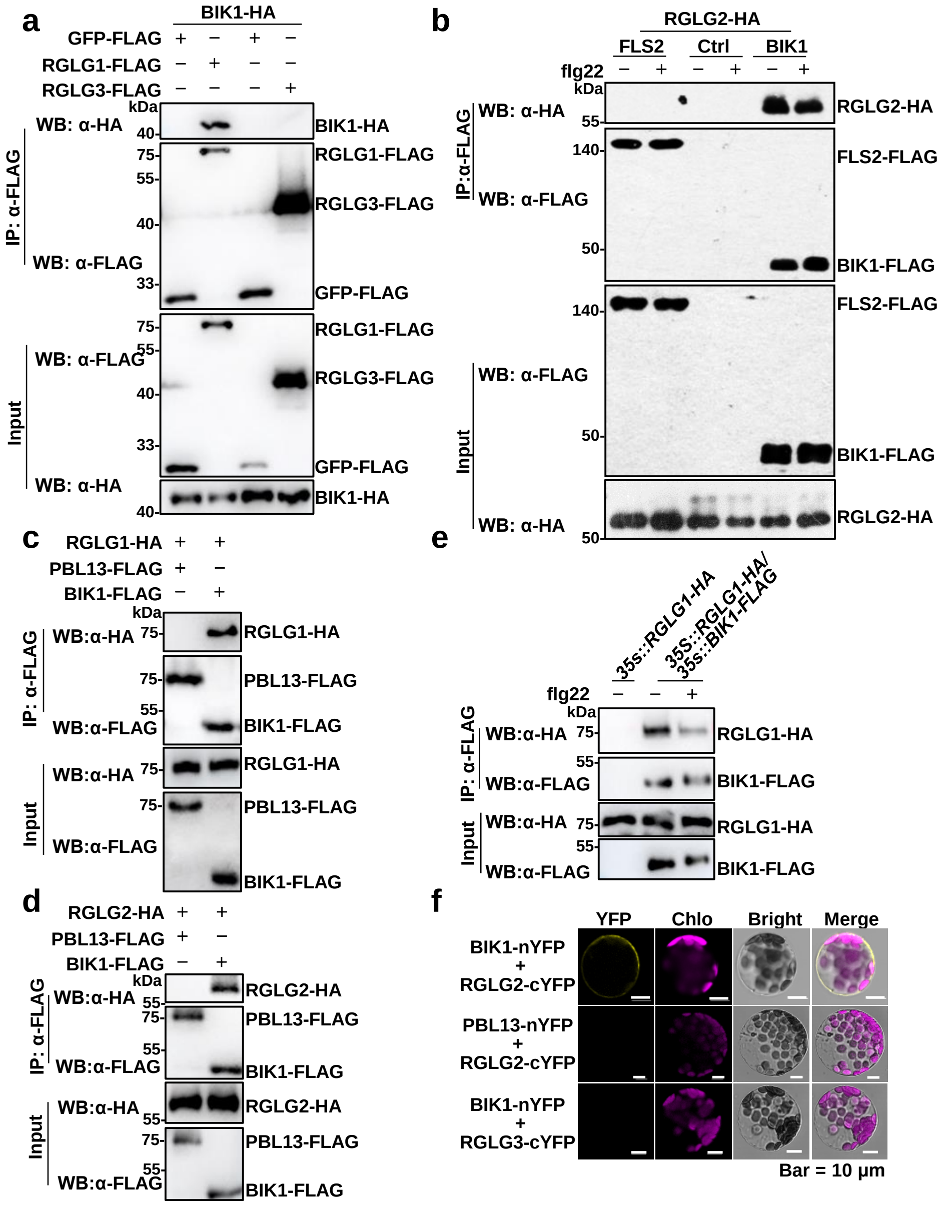


**Supplementary Fig. 7 RGLG1/2 specifically associate with BIK1.** **a**, RGLG3 does not associate with BIK1. BIK1-HA was co-expressed with GFP-FLAG, RGLG1-FLAG, or RGLG3-FLAG in Arabidopsis protoplasts. IPs were performed using anti-FLAG antibodies. **b**, RGLG2 does not associate with FLS2. RGLG2-HA was expressed together with FLS2-FLAG or BIK1-FLAG in Arabidopsis protoplasts. Protoplasts were treated with or without 2.5 μM flg22 for 30 min. IPs were performed using anti-FLAG antibodies. **c** and **d**, RGLG1/2 are not associated with PBL13. PBL13-FLAG was expressed with RGLG1/2-HA in Arabidopsis protoplasts. **e**, RGLG1 is associated with BIK1 in stable transgenic plants. *p35S::RGLG1-HA/p35S::BIK1-FLAG* and *p35S::RGLG1-HA*/Col-0 seedlings were treated with 5 μM flg22 for 30 min. BIK1-FLAG was immunoprecipitated with anti-FLAG antibodies. **f**, RGLG2 associates with BIK1 at the plasma membrane. The indicated BiFC constructs were transfected into Arabidopsis protoplasts, and fluorescence was visualized by confocal microscopy. Scale bars: 10 μm. **a**-**f**, images shown are representative of at least two independent experiments.

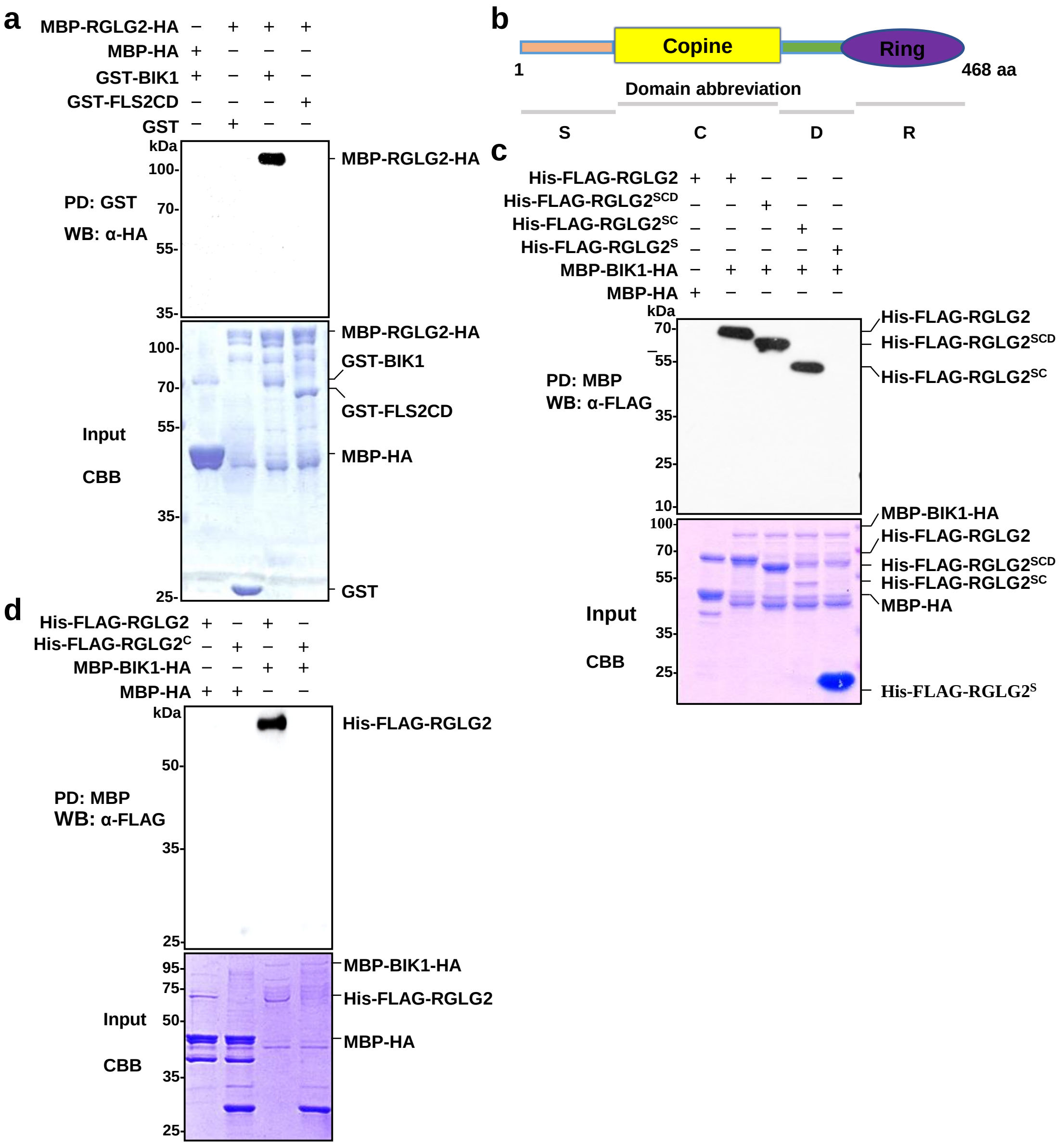


**Supplementary Fig. 8 RGLG2 directly interacts with BIK1 but not FLS2CD. a**, RGLG2 directly interacts with BIK1. The recombinant GST-BIK1/GST-FLS2CD/GST proteins immobilized on GSH beads were incubated with purified MBP-RGLG2-HA/MBP-HA proteins. The pulled-down MBP-RGLG2-HA proteins were detected by immunoblotting with anti-HA. All input proteins were separated by SDS-PAGE and stained with Coomassie Brilliant Blue (CBB). Images shown are representative of two independent experiments. **b**, Schematic representation of RGLG2 protein. The region at the N-terminus is called S domain, the region lying between the Copine (C) and the RING (R) domains is called D domain. **c** and **d**, The S-C domains of RGLG2 are responsible for the interaction of RGLG2 with BIK1. The recombinant MBP-BIK1-HA or MBP-HA proteins immobilized on amylose resin were incubated with His-FLAG-RGLG2 and different truncated RGLG2 variants. MBP pull-down was performed and the pulled-down proteins were detected by immunoblotting with anti-FLAG antibodies. Images shown are representative of two independent experiments.

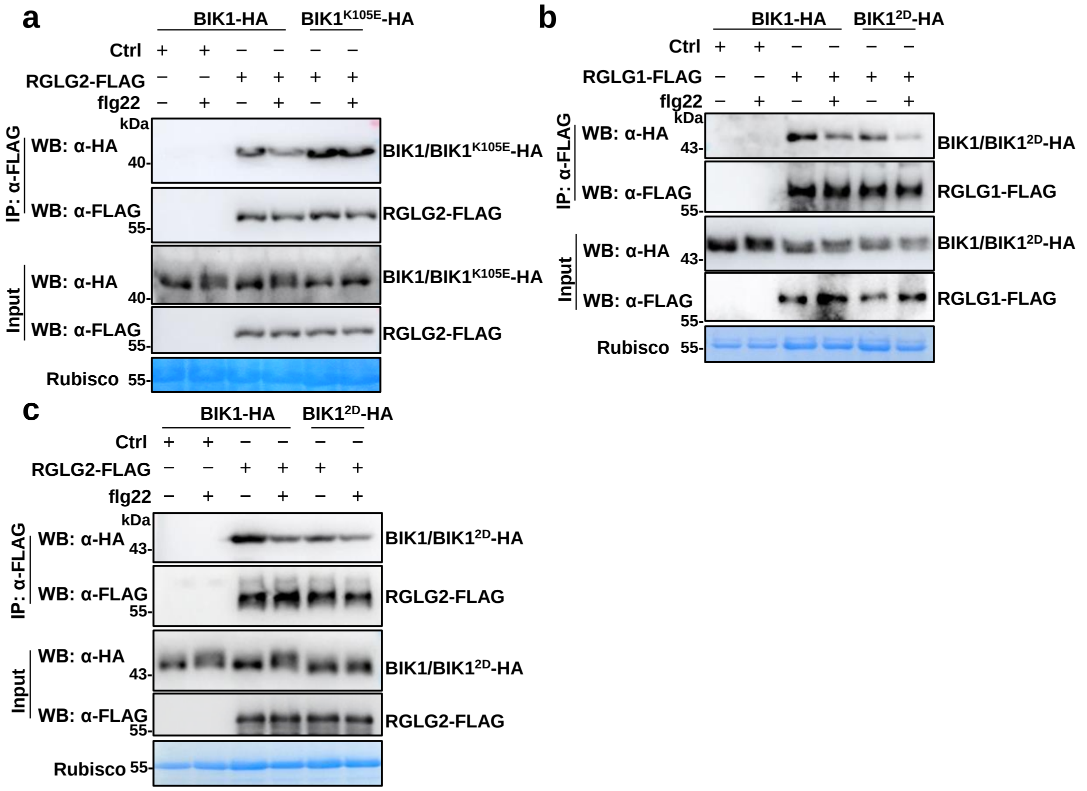


**Supplementary Fig. 9 RGLG1/2 preferentially associate with the hypo-phosphorylated BIK1. a**, Flg22 treatment does not affect the association between RGLG2 and $BIK1^{K105E}$. RGLG2-FLAG and BIK1-HA or $BIK1^{K105E}$-HA were co-expressed in protoplasts. Protoplasts were treated with or without 2.5 μM flg22 for 30 min. RGLG2-FLAG was immunoprecipitated with anti-FLAG antibodies. Images shown are representative of three independent experiments. **b** and **c**, The association of RGLG1/2 with $BIK1^{2D}$ is weaker than with WT BIK1. RGLG1/2-FLAG and BIK1-HA or $BIK1^{2D}$ (BIK1S236D/T237D)-HA were co-expressed in protoplasts. Protoplasts were treated with or without 2.5 μM flg22 for 30 min. Images shown are representative of at least two independent experiments.

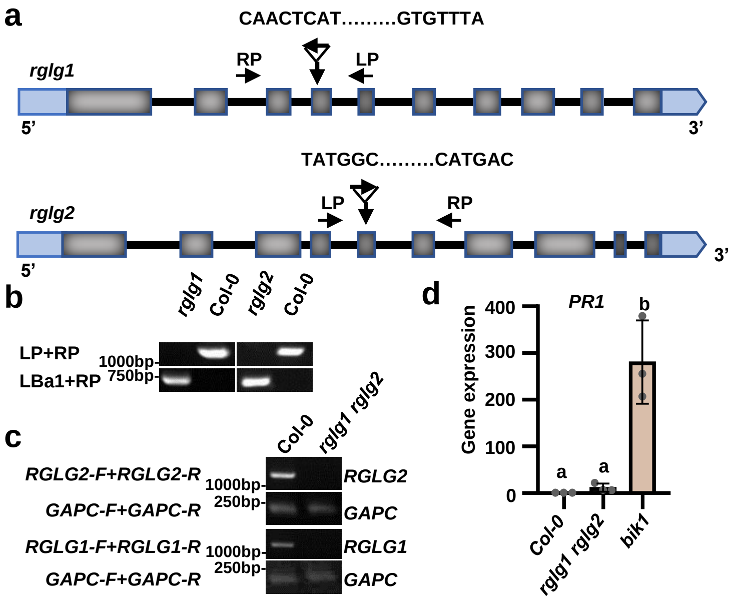


**Supplementary Fig. 10 Generation of *rglg1 rglg2* double mutant. a**, Schematic diagram of *RGLG1* and *RGLG2* gene structures. Gray boxes and blue boxes indicate exons and UTRs, respectively. The approximate positions of primers for genotyping are also shown. **b**, Identification of the homozygous T-DNA insertion mutants *rglg1* and *rglg2*. The gene specific primers and T-DNA-specific primers were used for genotyping. **c**, Measurement of the transcripts of *RGLG1* and *RGLG2* in *rglg1 rglg2* mutants by RT-PCR, *GAPC* was used as the internal control. **d**, Measurement of *PR1* transcript levels in *rglg1 rglg2*, *bik1*, and Col-0 plants. Total RNA was isolated from 12-day-old Arabidopsis seedlings. *PR1* transcript levels were analyzed by RT-qPCR and were then normalized to that of *GAPC*. Values represent the means ± SD of three independent replicates using independent seedling samples grown under the same conditions. Lowercase letters indicate significant differences with $P = 0.0009$ (one-way ANOVA with Tukey's multiple comparisons test). Source data are provided as a Source Data file (Supplementary dataset 4).

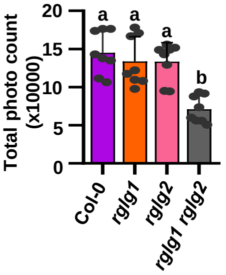


**Supplementary Fig. 11 The *rglg1 rglg2* double mutant produces less flg22-induced ROS than Col-0.** Five-week-old plants were treated with 200 nM flg22. ROS production was calculated as total relative light unit (RLU) (within 50 min of flg22 treatment). The values were shown as means $\pm$ SD ($n$ = 8 leave discs). Different letters denote significance difference (one-way ANOVA, $P < 0.0001$). Source data are provided as a Source Data file (Supplementary dataset 4).

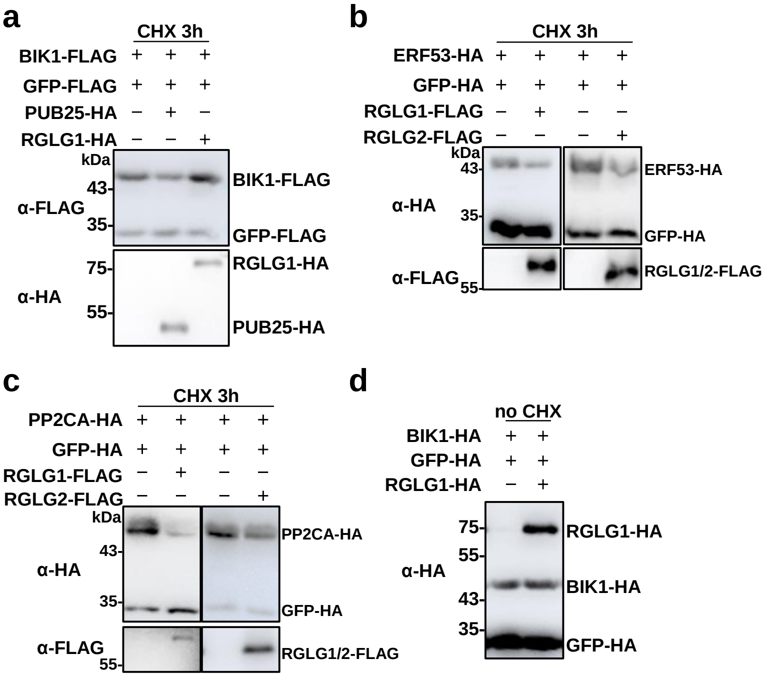


**Supplementary Fig. 12 RGLG1 promotes BIK1 protein accumulation.** **a**, RGLG1 expression promotes BIK1 protein accumulation in protoplasts. BIK1-FLAG was co-expressed with RGLG1-HA or PUB25-HA in protoplasts. GFP-FLAG was used as an internal transfection control. The protoplasts were treated with 50 μM CHX for 3 h before harvesting. The transiently expressed proteins were detected by immunoblotting with the corresponding antibodies. **b** and **c**, RGLG1/2 expression reduces ERF53 and PP2CA protein accumulation. RGLG1/2-FLAG were co-expressed with ERF53-HA or PP2CA-HA in protoplasts. GFP-HA was used as an internal transfection control. The protoplasts were treated with 50 μM CHX for 3 h before harvesting. **d**, Overexpression of RGLG1 hardly affects the BIK1 protein accumulation in protoplasts in the absence of CHX. BIK1-HA was co-expressed with RGLG1-HA in protoplasts. GFP-HA was used as an internal transfection control. **a**-**d**, images shown are representative of at least two independent experiments.

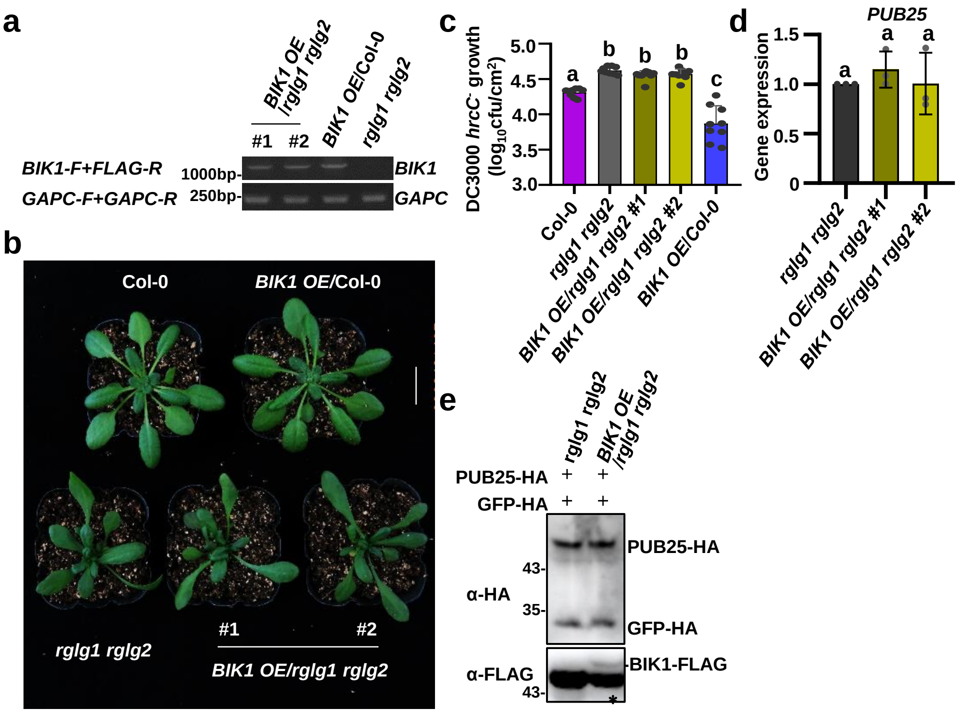


**Supplementary Fig. 13 BIK1 protein accumulation largely depends on RGLG1/2.** **a**, Measurement of mRNA levels of *BIK1-FLAG* in *p35S::BIK1-FLAG* (*BIK1* OE)/*rglg1 rglg2* (#1, #2) and *p35S::BIK1-FLAG* (*BIK1* OE)/Col-0 transgenic plants by RT-PCR. *GAPC* was used as an internal control. **b**, The growth phenotypes of *rglg1 rglg2*, *BIK1* OE/*rglg1 rglg2*, *BIK1* OE/Col-0, and Col-0 plants at 30 d post germination under a 12 h light/12 h dark cycle. Scale bar = 1 cm. **c**, Overexpressing *BIK1* in *rglg1 rglg2* only slightly restores the plant resistance to *Pst* DC3000 *hrcC*⁻. The leaves of 30-d-old plants were infiltrated with *Pst* DC3000 *hrcC*⁻ at the OD600 of 0.001, and bacterial growth was assessed 3 days post inoculation and was evaluated as colony-forming units per $cm^2$ of leaf area (CFU/$cm^2$). Individual data points were shown with means ± SD ($n$ = 9 leaves from three biological replicates). Different letters denote significance difference (One-way ANOVA, $P < 0.0001$). Source data are provided as a Source Data file (Supplementary dataset 4). **d**, Measurement of *PUB25* transcript levels. Total RNA was isolated from 30-day-old *rglg1 rglg2* and *BIK1* OE/*rglg1 rglg2* plants. *PUB25* transcript levels were analyzed by RT-qPCR and were then normalized to that of GAPC. Values represent the means ± SD of three independent replicates using independent seedling samples grown under the same conditions. $P$ = 0.6430 (one-way ANOVA with Tukey's multiple comparisons test). Source data are provided as a Source Data file (Supplementary dataset 4). **e**, PUB25-HA protein accumulation is not increased by *BIK1* overexpression. PUB25-HA was expressed in protoplasts isolated from *rglg1 rglg2* and *BIK1* OE/*rglg1 rglg2* plants. GFP-HA was used as an internal transfection control. BIK1-FLAG proteins in the transgenic plants were detected with anti-FLAG antibodies before the plants were used for protoplast isolation. The asterisk indicates the unspecific proteins detected with anti-FLAG antibodies. Images shown are representative of two independent experiments.

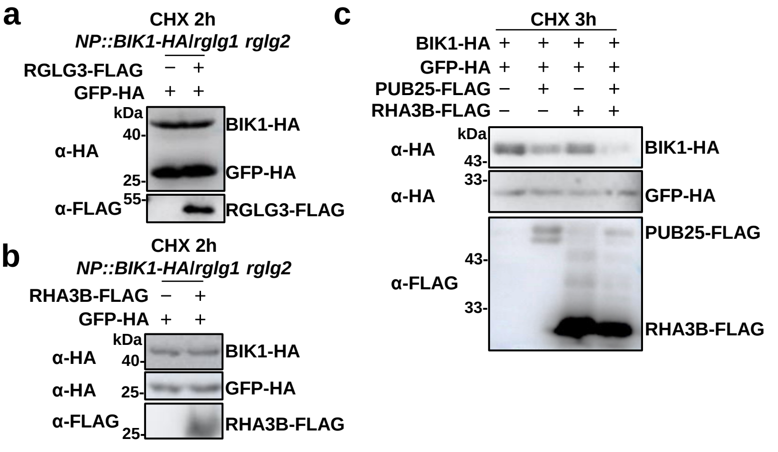


**Supplementary Fig. 14 RGLG3 and RHA3B do not promote BIK1 protein accumulation. a**, RGLG3 does not promote BIK1 protein accumulation. RGLG3-FLAG and GFP-HA were co-expressed in *pBIK1*(*NP*)*::BIK1-HA*/*rglg1 rglg2* protoplasts. The protoplasts were treated with 50 μM CHX for 2 h before harvesting. BIK1-HA proteins were detected by immunoblotting with anti-HA antibodies. GFP-FLAG was used as an internal transfection control. **b**, RHA3B does not promote BIK1 protein accumulation. *RHA3B-FLAG* and *GFP-HA* were expressed in *NP::BIK1-HA*/*rglg1 rglg2* protoplasts. The protoplasts were treated with 50 μM CHX for 2 h before harvesting. **c**, RHA3B does not affect the PUB25-mediated BIK1 degradation. BIK1-HA, PUB25-FLAG, and GFP-HA were expressed with or without RHA3B-FLAG in Arabidopsis protoplasts. The protoplasts were treated with 50 μM CHX for 3 h before harvesting. BIK1-HA proteins were detected with immunoblotting with anti-HA antibodies. RHA3B-FLAG and PUB25-FLAG proteins were detected with anti-FLAG antibodies. GFP-HA was used as an internal transfection control. **a-c**, images shown are representative of three independent experiments.

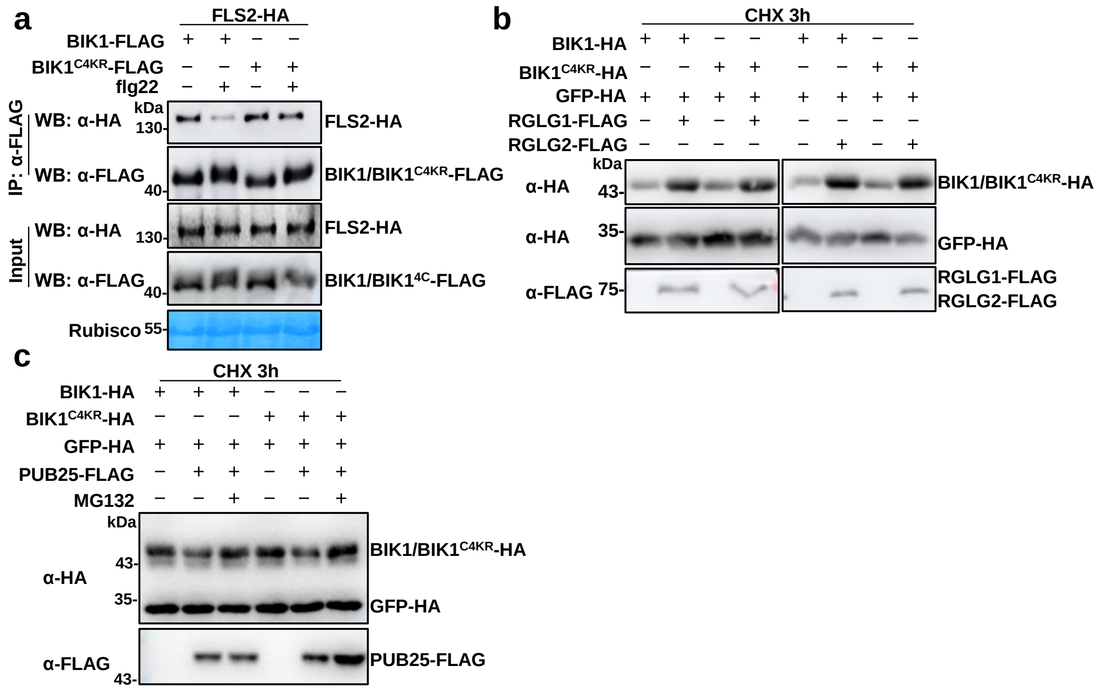


**Supplementary Fig. 15 The role of RGLG1/2 in regulating BIK1 is distinct from that of RHA3A/B. a**, BIK1$^{C4KR}$ does not dissociate from FLS2 upon flg22 treatment. FLS2-HA was co-expressed with BIK1-FLAG or BIK1$^{C4KR}$-FLAG in Arabidopsis protoplasts. Protoplasts were treated with or without 2.5 μM flg22 for 30 min. Co-IP was performed with the indicated antibodies. **b**, Protein accumulation of BIK1$^{C4KR}$ promoted by RGLG1/2 is comparable to that of WT BIK1. BIK1-HA or BIK1$^{C4KR}$-HA was co-expressed with RGLG1/2-FLAG in protoplasts. GFP-FLAG was used as an internal transfection control. The protoplasts were treated with 50 μM CHX for 3 h before harvesting. The transiently expressed proteins were detected by immunoblotting with the corresponding antibodies. **c**, PUB25 mediates the degradation of BIK1$^{C4KR}$ as it does for WT BIK1. BIK1$^{C4KR}$-HA was co-expressed with PUB25-FLAG in protoplasts. GFP-FLAG was used as an internal transfection control. The protoplasts were treated with 50 μM CHX, and with or without 30 μM MG132 for 3 h before harvesting. **a**-**c**, images shown are representative of at least two independent experiments.

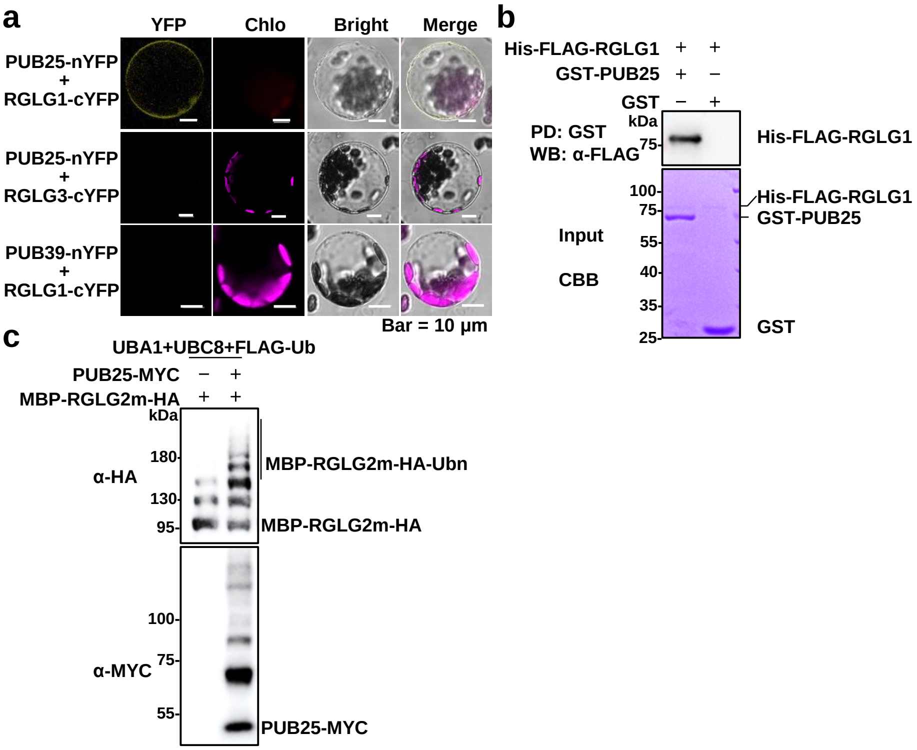


**Supplementary Fig. 16 PUB25 interacts RGLG1 and mediates RGLG2m ubiquitination.** **a**, PUB25 is associated with RGLG1 at the plasma membrane. The indicated BiFC constructs were transfected into Arabidopsis protoplasts, and fluorescence was visualized by confocal microscopy. Scale bars: 10 μm. Images shown are representative of at least two independent experiments. **b**, RGLG1 directly interacts with PUB25 *in vitro*. The recombinant GST-PUB25/GST proteins immobilized on GSH beads were incubated with purified His-FLAG-RGLG1 proteins. The pulled-down His-FLAG-RGLG1 proteins were detected by immunoblotting with anti-FLAG antibodies. **c**, PUB25 ubiquitinates RGLG2m *in vitro*. pACYC-PUB25-MYC-AtUBC8-S, pCDF-MBP-RGLG2m-HA-AtUBA1-S, and pET-His-FLAG-Ub were co-transformed in *E. coli*. The bacterial lysates were subjected to immunoblotting analysis with anti-HA antibodies for detecting RGLG2m ubiquitination or with anti-MYC antibodies for detecting PUB25 autoubiquitination. **b** and **c**, images shown are representative of three independent experiments.

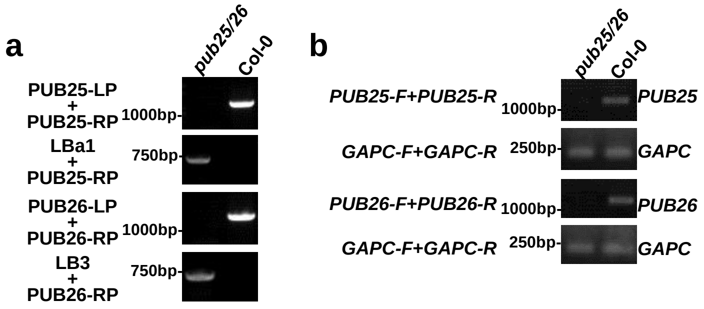


**Supplementary Fig. 17 Identification of the homozygous *pub25 pub26* double mutant. a**, Genotyping analysis of the *pub25 pub26* double mutant. The gene specific primers and T-DNA-specific primers were used for genotyping. **b**, Measurement of the transcripts of *PUB25* and *PUB26* in *rglg1 rglg2* mutants by RT-PCR, *GAPC* was used as the internal control.

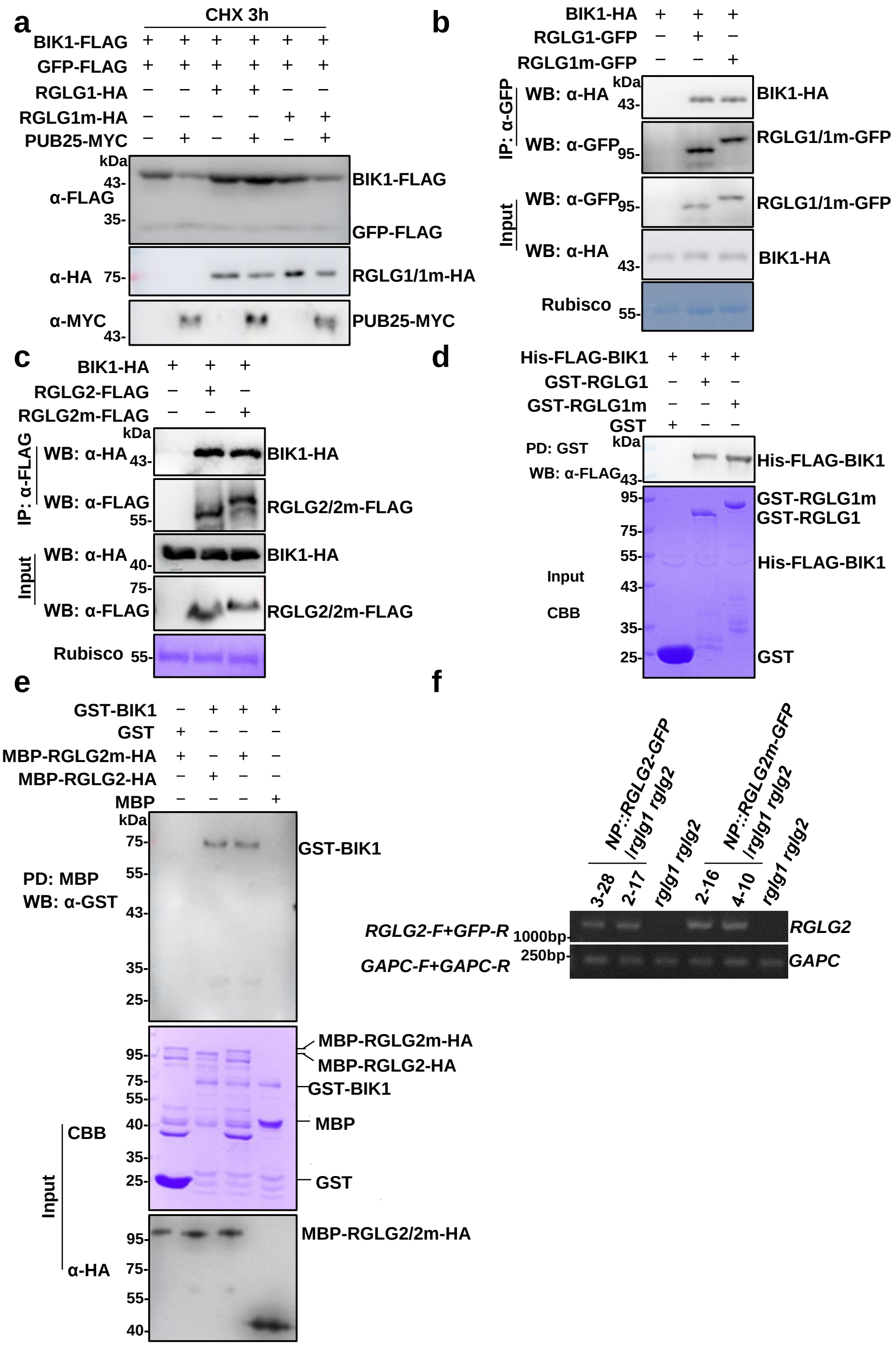


**Supplementary Fig. 18 The ubiquitin ligase activity is required for the role of RGLG1/2 in regulating immunity.** **a**, RGLG1m does not suppress the PUB25-mediated BIK1 protein degradation. BIK1-FLAG and GFP-FLAG were co-expressed together with or without RGLG1/RGLG1m-HA or PUB25-MYC in Arabidopsis protoplasts. GFP-FLAG was used as an internal transfection control. The protoplasts were treated with 50 μM CHX for 3 h before harvesting. The transiently expressed proteins were detected by immunoblotting with the corresponding antibodies. **b** and **c**, The association of BIK1 with RGLG1/2 was comparable to that with RGLG1m/2m *in vivo*. BIK1-HA was co-expressed with RGLG1/RGLG1m-GFP or RGLG2/RGLG2m-FLAG in Arabidopsis protoplasts. IP was performed as indicated. **d** and **e**, The interaction of BIK1 with RGLG1/2 was comparable to that with RGLG1m/2m *in vitro*. (**d**) The recombinant GST-RGLG1/GST-RGLG1m/GST proteins immobilized on GSH beads were incubated with purified His-FLAG-BIK1 proteins. The pulled-down His-FLAG-BIK1 proteins were detected by immunoblotting with anti-FLAG antibodies. (**e**) Recombinant MBP-HA, MBP-RGLG2-HA, or MBP-RGLG2m-HA proteins immobilized on amylose resin were incubated with GST-BIK1 proteins, and MBP pulled-down assays were performed. **a**-**e**, images shown are representative of at least two independent experiments. **f**, Detection of *RGLG2/RGLG2m-GFP* mRNA in *rglg1 rglg2* complementation lines. *RGLG2/RGLG2m-GFP* transcripts were detected by RT-PCR in *pRGLG2::RGLG2m-GFP*/*rglg1 rglg2* and *pRGLG2::RGLG2-GFP*/*rglg1 rglg2* transgenic lines. *GAPC* mRNA was detected as an internal control.

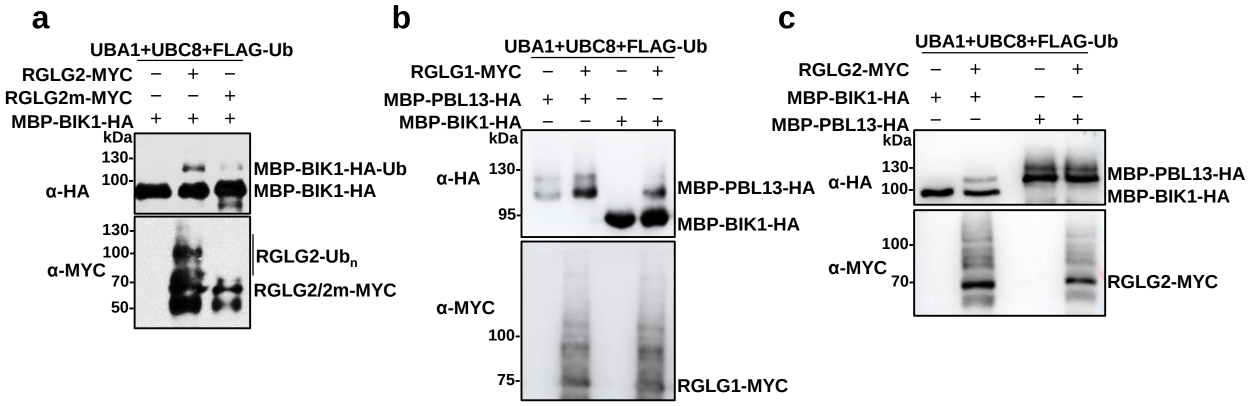


**Supplementary Fig. 19 RGLG1/2 specifically ubiquitinate BIK1.** **a**, RGLG2m is not able to ubiquitinate BIK1. pACYC-RGLG2/RGLG2m-MYC-AtUBC8-S, pCDF-MBP-BIK1-HA-AtUBA1-S, and pET-His-FLAG-Ub were co-expressed in *E. coli*. The bacterial lysates were subjected to immunoblotting analysis with anti-HA antibodies for detecting BIK1 ubiquitination or with α-MYC antibodies for detecting RGLG2/RGLG2m autoubiquitination. **b** and **c**, RGLG1/2 does not ubiquitinate PBL13. pACYC-RGLG1/2-MYC-AtUBC8-S, pCDF-MBP-PBL13-HA-AtUBA1-S, and pET-His-FLAG-Ub were co-transformed in *E. coli*. The bacterial lysates were subjected to immunoblotting analysis. **a**-**c**, images shown are representative of at least two independent experiments.

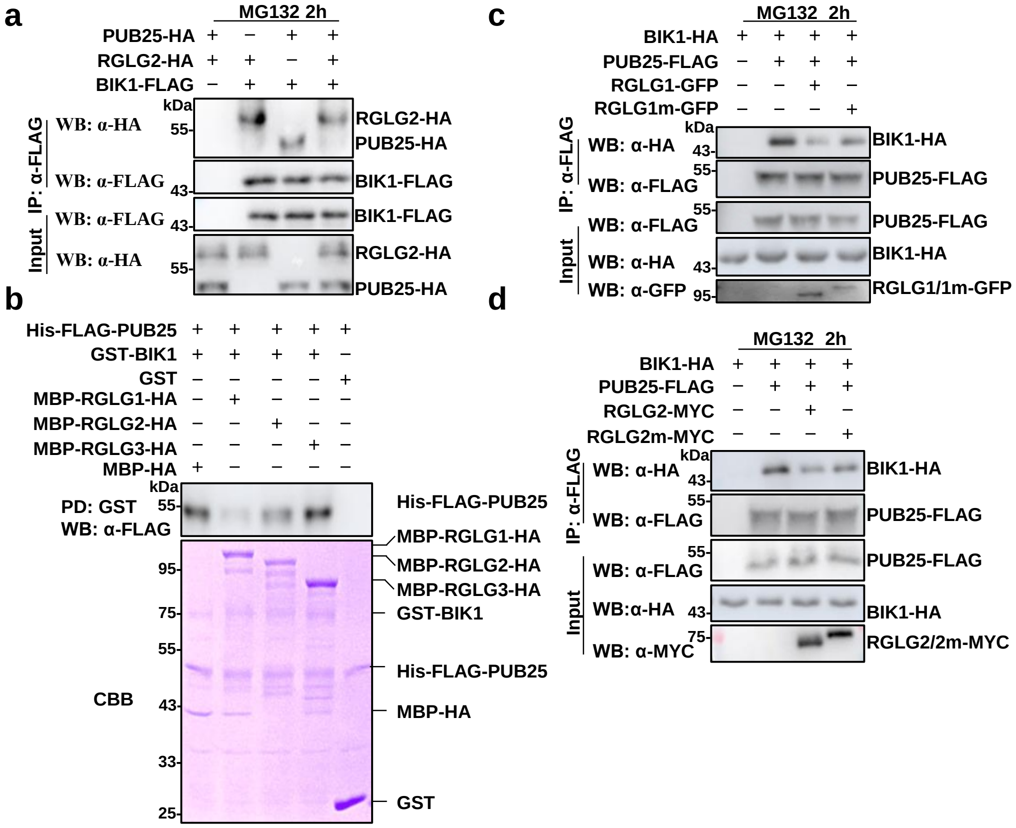


**Supplementary Fig. 20 RGLG1/2 compete with PUB25 for interacting with BIK1.** **a**, RGLG2 suppresses the association of PUB25 and BIK1. BIK1-FLAG and PUB25-HA were expressed together with or without RGLG2-HA in Arabidopsis protoplasts. IPs were performed using anti-FLAG antibodies, the associated proteins were detected by immunoblotting with anti-HA antibodies. Protoplasts were treatment with 30 μM MG132 for 2 h before harvesting. **b**, RGLG1/2, but not RGLG3 reduce the interaction between BIK1 and PUB25 *in vitro*. The recombinant proteins were expressed in *E. coli* and were affinity purified, GST-BIK1 proteins immobilized on GST beads were incubated with purified MBP-RGLG1/2/3-HA and His-FLAG-PUB25 proteins with gentle shaking. GST pull-down was performed and the pulled-down His-FLAG-PUB25 proteins were detected by immunoblotting with anti-FLAG antibodies. Input proteins were separated on SDS-PAGE and stained with CBB. **a** and **b**, images shown are representative of three independent experiments. **c** and **d**, The association of BIK1 and PUB25 is also reduced by RGLG1m/2m expression. BIK1-HA and PUB25-FLAG were expressed together with RGLG1/1m-GFP or RGLG2/2m-MYC in Arabidopsis protoplasts. Protoplasts were treatment with 30 μM MG132 for 2 h before harvesting. IPs were performed using anti-FLAG antibodies. Images shown are representative of at least two independent experiments.

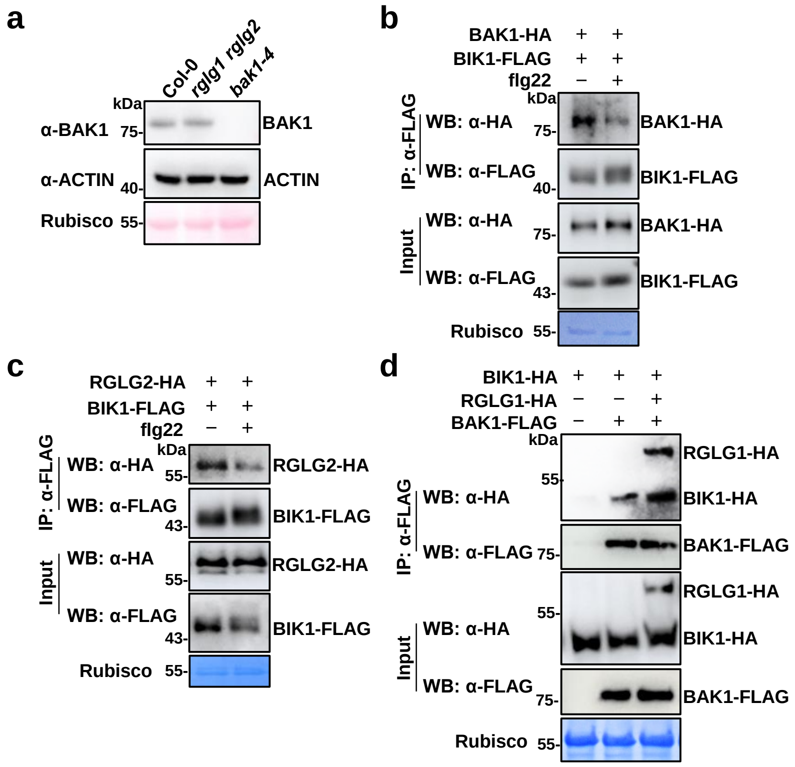


**Supplementary Fig. 21 RGLG1 promotes the association of BAK1 and BIK1. a**, BAK1 protein level is comparable in *rglg1 rglg2* and Col-0 plants. Total proteins were isolated from 7-d-old *rglg1 rglg2*, Col-0, and *bak1-4* seedlings (15 seedlings per sample). BAK1 proteins were detected by immunoblotting with anti-BAK1 antibodies. ACTIN was used as a loading control. Images shown are representative of two independent experiments. **b** and **c**, The association of BAK1-BIK1 or RGLG2-BIK1 is reduced upon flg22 treatment. BIK1-FLAG was co-expressed BAK1-HA or RGLG2-HA in Arabidopsis protoplasts. The protoplasts were treated with 2.5 μM flg22 for 30 min. BIK1-FLAG was immunoprecipitated with anti-FLAG antibodies. **d**, BAK1, BIK1, and RGLG1 are in the same complex. BAK1-FLAG was co-expressed with BIK1-HA and RGLG1-HA in protoplasts. BAK1-FLAG proteins were immunoprecipitated with anti-FLAG antibodies. **b** and **c**, images shown are representative of two independent experiments.

**Supplementary Table 1. ANOVA tables.**

**Sum Sq = Sum of squares; df = degrees of freedom; Mean Sq = Mean Squares**

| Figure Number | | Sum Sq | df | Mean Sq | F-Val | P-Value |
|---|---|---|---|---|---|---|
| Figure 2a | Treat | 1.960e+015 | | 6.534e+014 | 21.90 | 0.0003 |
| | | 2.387e+014 | | 29839680654 | | |
| | | 2.199e+015 | | | | |
| Figure 3a | Treat | 30447354 | 3 | 10149118 | 5.465 | 0.0013 |
| | Resid | 363984948 | 196 | 1857066 | | |
| | Total | 394432302 | 199 | | | |
| Figure 5c | Treat | 6.397 | 5 | 1.279 | 25.08 | < 0.0001 |
| | Resid | 2.449 | 48 | 0.05101 | | |
| | Total | 8.846 | 53 | | | |
| Figure 5d | Treat | 15811295610 | 5 | 3162259122 | 11.01 | < 0.0001 |
| | Resid | 12067105988 | 42 | 287312047 | | |
| | Total | 27878401598 | 47 | | | |
| Supplementary fig.1b At3G25250 | Treat | 42810 | 7 | 6116 | 37.32 | < 0.0001 |
| | Resid | 2622 | 16 | 163.9 | | |
| | Total | 45432 | 23 | | | |
| Supplementary fig.1b At2G19190 | Treat | 24779 | 7 | 3540 | 8.266 | 0.0003 |
| | Resid | 6852 | 16 | 428.3 | | |
| | Total | 31631 | 23 | | | |
| Supplementary fig.1b At5G24110 | Treat | 2141176 | 7 | 305882 | 21.52 | < 0.0001 |
| | Resid | 227448 | 16 | 14215 | | |
| | Total | 2368624 | 23 | | | |
| Supplementary fig.1b At1G51890 | Treat | 13237 | 7 | 1891 | 6.686 | 0.0008 |
| | Resid | 4525 | 16 | 282.8 | | |
| | Total | 17762 | 23 | | | |
| Supplementary fig.1b At2G35980 | Treat | 41511 | 7 | 5930 | 18.48 | < 0.0001 |
| | Resid | 5135 | 16 | 320.9 | | |
| | Total | 46645 | 23 | | | |
| Supplementary fig.1b At2G38470 | Treat | 3414 | 7 | 487.7 | 16.33 | < 0.0001 |
| | Resid | 477.8 | 16 | 29.86 | | |
| | Total | 3892 | 23 | | | |
| Supplementary fig.6a | Treat | 2.063e+014 | 2 | 1.032e+014 | 31.91 | 0.0006 |
| | Resid | 193958840215 | 6 | 32326473369 | | |
| | Total | 2.257e+014 | 8 | | | |
| Supplementary fig.6b | Treat | 1.655e+014 | 2 | 82761361702 | 22.18 | 0.0017 |
| | Resid | 223842712184 | 6 | 37307118697 | | |
| | Total | 1.879e+014 | 8 | | | |
| Supplementary fig.10d | Treat | 150795 | 2 | 75397 | 28.33 | 0.0009 |
| | Resid | 15966 | 6 | 2661 | | |
| | Total | 166761 | 8 | | | |
| Supplementary fig.11 | Treat | 27055982950 | 3 | 9018660983 | 12.93 | < 0.0001 |
| | Resid | 19536529821 | 28 | 697733208 | | |
| | Total | 46592512771 | 31 | | | |
| Supplementary fig.13c | Treat | 3.509 | 4 | 0.8771 | 55.69 | < 0.0001 |
| | Resid | 0.6301 | 40 | 0.01575 | | |
| | Total | 4.139 | 44 | | | |
| Supplementary fig.13d | Treat | 0.04167 | 2 | 0.02084 | 0.475 | 0.6430 |
| | Resid | 0.2628 | 6 | 0.04380 | | |
| | Total | 0.3045 | 8 | | | |

**Supplementary Table 2. Student's *t*-test tables.**

| Figure Number | | P-Value | T | df | One- or two-tailed P value? |
|---|---|---|---|---|---|
| Figure 3c | *rglg1rglg2* | 0.0018 | 3.540 | 22 | Two-tailed |
| Figure 3d | *rglg1rglg2* | <0.0001 | 7.676 | 22 | Two-tailed |
