# Supplementary material for: BIK1 protein homeostasis is maintained by the interplay of different ubiquitin ligases in immune signaling
Source: Nat Commun. 2023 Aug 2;14:4624. doi: 10.1038/s41467-023-40364-0 (PMC10397244; doi:10.1038/s41467-023-40364-0)
Supplement: Supplementary file 3 — Description of Additional Supplementary Files [file 41467_2023_40364_MOESM3_ESM.pdf]

Description of Additional Supplementary Files

Supplementary Data 1. RNA-seq analysis of dynamic gene expression in response to persistent flg22 treatment. Twelve-day-old Arabidopsis seedlings were treated with 5 µM flg22 for 0, 15, 30, 60, 120, 240, 360, 480 min. The deferentially expressed genes (DEGs) were identified by comparison with time 0 via the R package DESeq2 [$\log_2$(fold change, FC) > 1, $P_{adj} < 0.05$].

Supplementary Data 2. The FPKM values of the 179 ubiquitin ligase genes with the expression pattern #2 upon persistent flg22 treatment. Four primary expression patterns (Pattern #1-4) were obtained based on the time-course gene expression patterns analyzed by STEM software.

Supplementary Data 3. Sequences of oligonucleotides used in this work.
